# The terrestrial microsnail genus *Aulacospira* Möllendorff, 1890 (Eupulmonata, Stylommatophora, Hypselostomatidae) in Thailand with key to Thai species

**DOI:** 10.3897/zookeys.980.54100

**Published:** 2020-10-28

**Authors:** Pongrat Dumrongrojwattana, Kitti Tanmuangpak

**Affiliations:** 1 Department of Biology, Faculty of Science, Burapha University, Bangsaen, Chonburi 20131 Thailand Burapha University Chon Buri Thailand; 2 Program of Biology, Department of Science, Faculty of Science and Technology, Loei Rajabhat University, Loei 42000 Thailand Loei Rajabhat University Loei Thailand

**Keywords:** new species, taxonomy

## Abstract

Thai terrestrial microsnails in the genus *Aulacospira* Möllendorff, 1890 are revised based on the collection of the Zoological Research Collection, Burapha University, Chonburi Province, Thailand and recently collected material. Three new species are described: *Aulacospira nutadhirai* sp.nov. from Southern Thailand, and *Aulacospira tekavongae***sp.nov.** and *Aulacospira vanwalleghemi***sp. nov.** from Eastern Thailand. The radula and genital system are described, and a key to Thai species is presented.

## Introduction

Microsnails in the genus *Aulacospira* Möllendorff, 1890 were first described by Möllendorff in 1890 from the Philippines where there are six species: *A. hololoma* (Möllendorff, 1887), *A. mucronata* (Möllendorff, 1887), *A. scalatella* (Möllendorff, 1888), *A. porrecta* Quadras & Möllendorff, 1894, *A. triptycha* Quadras & Möllendorff, 1895 and *A. rhombostoma* Quadras & Möllendorff, 1896. Two species, *A. lampangensis* Panha & Burch, 2001 and *A. smaesarnensis* Panha & Burch, 2001 were described from Thailand by Panha and Burch (2001), but subsequent work by Panha et al. (2004), Dumrongrojwattana and Panha (2005, 2006), and Dumrongrojwattana (2008) increased the number of species to seven with the addition of *A. pluangtong* Panha & Burch, 2004 , *A. khaopratun* Dumrongrojwattana & Panha, 2005, *A. khaobote* Dumrongrojwattana & Panha, 2006, *A. depressa* Dumrongrojwattana & Panha, 2006, and *A. panhai* Dumrongrojwattana, 2008. Of the known Thailand species, only *A. lampangensis* is known from the north of the country and all other species are restricted to eastern Thailand (Panha and Burch 2005, Dumrongrojwattana and Panha 2006, Dumrongrojwattana, 2008) (Table 1). Most of previous taxonomic studies on *Aulacospira* were based only on shell morphology (Pilsbry 1916–1918; Panha et al. 2004; Panha and Burch 2005; Dumrongrojwattana and Panha 2005, 2006; Dumrongrojwattana 2008; Vermeulen et al. 2007; Páll-Gergely et al. 2019), and there is no information on the radula and genital system. We provide an overview of the Thai *Aulacospria* and describe three new species from eastern and southern Thailand based on the shell morphology, radula, and genital system. A key to Thai species is presented.

**Table 1.** Species list, diagnostic characteristics and geographic distribution of *Aulacospira* species in Thailand.

Species	Diagnostic characteristics					Geographic distribution	References
	Shell morphology	No. of apertural teeth	W/H ratio	Radula formula	Genital system		
**With apertural teeth**							
*Aulacospira lampangensis*	Shell moderately high, whorl shouldered	4	1.44	–	–	Type locality only	1, 4
*Aulacospira panhai*	Shell semi-depressed, body whorl acutely angular	6	0.61	7–8:4:1:4:7–8	–	Chonburi and Rayong provinces	6, 8
*Aulacospira pluangtong*	Shell high, body whorl obtusely angular	4	1.06	7–8:4:1:4:7–8	Penis longer than epiphallus; vas deferens very long, slender; gametolytic sac very long, cylindrical.	Limestone hills in Bothong District, Chonburi Province	2, 4, 7
*Aulacospira smaesarnensis*	Shell high, body whorl acutely angular	3	1.15	7–8:4:1:4:7–8	–	Type locality only	1, 4, 7
**Without apertural teeth**							
*Aulacospira depressa*	Shell flattened, spire very low	none	2.60	7–8:4:1:4:7–8	–	Type locality only	5, 9
*Aulacospira khaobote*	Spire high, body whorl with deep spiral groove	none	1.21	–	–	Type locality only	5, 8, 9
*Aulacospira khaopratun*	Shell moderately high, body whorl with two spiral carinae	none	1.71	7–8:4:1:4:7–8	–	Chonburi, Rayong, and Srakeo provinces	3, 4, 9
*Aulacospira tekavongae* sp. nov.	Shell high, body whorl with deep spiral groove	none	0.98	7–8:4:1:4:7–8	Penis shorter than epiphallus; vas deferens long, slender; gametolytic sac long, cylindrical, with anterior and central portion bulging.	Type locality only	Present study
*Aulacospira nutadhirai* sp. nov.	Spire low, body whorl inflation with shallow spiral groove	none	1.61	7–8:4:1:4:7–8	Penis shorter than epiphallus; vas deferens short, slender; gametolytic sac long, cylindrical.	Type locality only	Present study
*Aulacospria vanwalleghemi* sp. nov.	Spire high, body whorl strongly keel at periphery	none	1.04	7–8:4:1:4:7–8	Penis shorter than epiphallus; vas deferens short, slender; gametolytic sac very long, slender.	Type locality only	Present study

*References: 1 = Panha and Burch 2001; 2 = Panha et al. 2004; 3 = Dumrongrojwattana and Panha 2005; 4 = Panha and Burch 2005; 5 = Dumrongrojwattana and Panha 2006; 6 = Dumrongrojwattana 2008; 7 = Boon-ngam et al. 2008; 8 = Chidchua and Dumrongorjwattana 2010; 9 = Inmadon et al. 2011.

## Materials and methods

**Figure 1.**
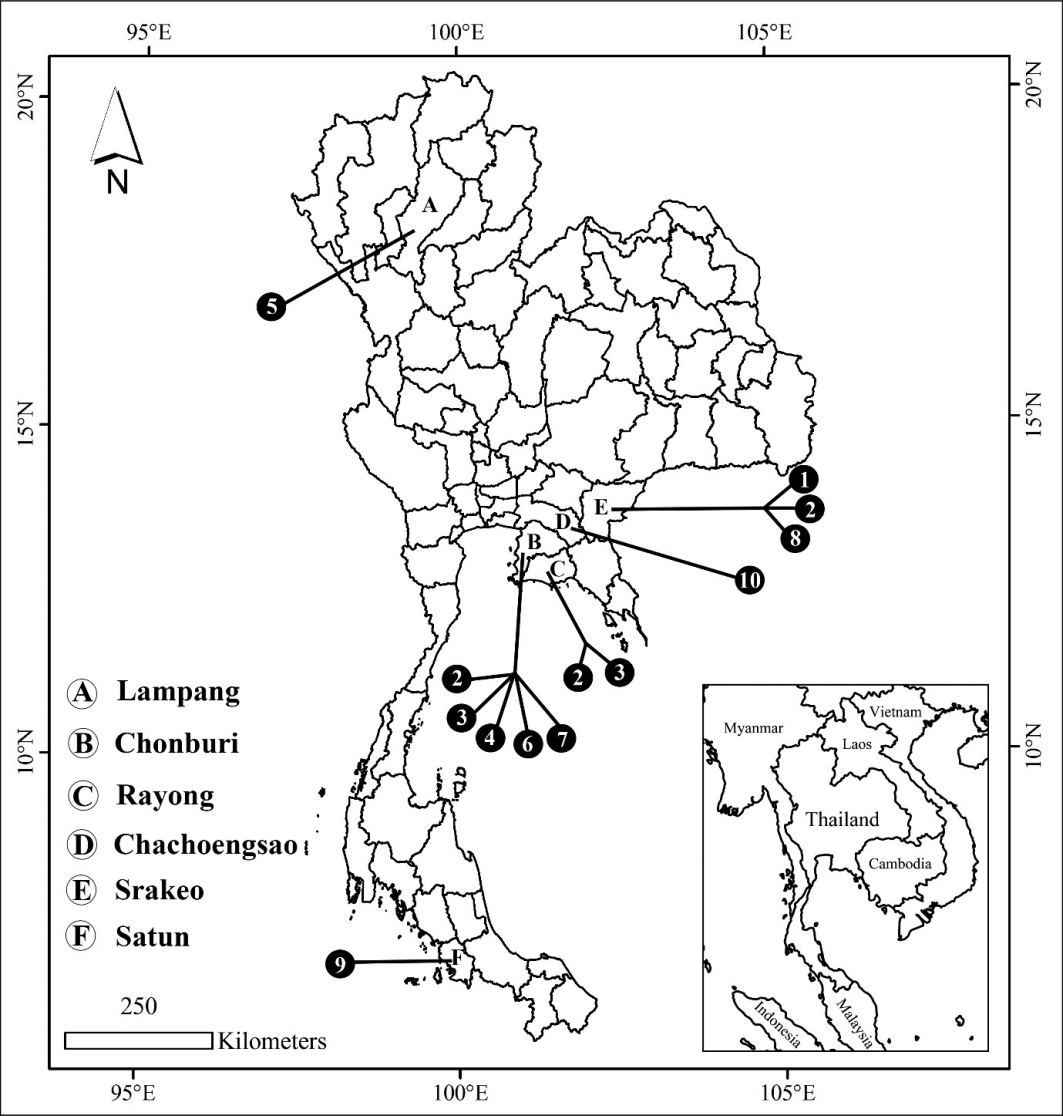
Distribution map of *Aulacospira* spp. in Thailand: **1***A. depressa***2***A. khaopratun***3***A. panhai***4***A. khaobote***5***A. lampangensis***6***A. smaesarnensis***7***A. pluangtong***8***Aulacospira tekavongae* sp. nov. **9***Aulacospira nudtadhirai* sp. nov. **10***Aulacospira vanwalleghemi* sp. nov.

Types and voucher specimens of previously described species were deposited in the reference collection of the Zoology Laboratory, Faculty of Science, Burapha University. The new species were collected from limestone hills in eastern Thailand. Collecting sites and their distribution shown in Figure 1. Shells were digitally photographed using a Cannon MP–E. Shell terminology (e.g., whorl number, apertural teeth, etc.) follows Panha and Burch (2005). Shell measurements (in mm) were taken by using a digital vernier caliper (Mitutoyo, Japan). Taxonomic identification of specimens was based mainly on Panha and Burch (2005), Dumrongrojwattana and Panha (2005, 2006), and Dumrongrojwattana (2008). Dichotomous key construction was based on shell morphology. The radula was exacted by boiling the dead snail in 1.0% NaOH for 1–2 minutes in a small test tube. The contents of the test tube were transfered into a small petri dish and radula removed under an Olympus SZ51 stereomicroscope. The radula was washed in three changes of distilled water, 3 minutes per rinse, and then dehydrated in an ethyl alcohol series of 10%, 30%, 50% and 70% v/v for 5 minutes in each concentration. The radula was then air-dried on a stub and scanned using an LEO 1450 VP scanning electron microscope at the Microscopic Center, Faculty of Science, Burapha University, Chonburi Province. Adult snails were also dissected to examine the genital system.

The following abbreviations are used in the text and figures: leg. = collected by, H = shell height, W = shell width, W/H = shell width/height ratio, ag = albumen gland, at = atrium, e = epiphallus, erc = epiphallic retractor caecum, fo = free oviduct, hd = hermaphroditic duct, p = penis, pro = prostate gland, gs = gametolytic sac, ut = uterus, v = vagina, vd = vas deferens.

Specimens were studied from the following collections:

**MNHN**Muséum national d’Histoire naturelle, Paris, France.

**NHLRU** Natural History Museum of Loei Rajabhat University, Loei, Thailand.

**THNHM** Thailand Natural History Museum, Pathum Thani, Thailand.

**ZRCBUU** Zoological Research Collection of Burapha University, Bangsaen, Thailand.

## Taxonomy

### Family Hypselostomatidae Zilch, 1959

Zilch 1959: 162, as Hypselostomatinae, subfamily of Chondrinidae.

Schileyko 1998: 136.

Bouchet et al. 2017: 43.

#### Genus Aulacospira

Taxon classificationAnimaliaStylommatophoraHypselostomatidae

Möllendorff, 1890

A32D7541-4B74-514C-A0EA-BC7FFAC764C1

Aulacospira Möllendorff 1890: 224; Páll-Gergely et al. 2019.
Micropetasus Möllendorff 1890: 224, as section Aulacospira; type species: Helix scalatella Möllendorff, 1888.

##### Type species.

*Helix scalatella* Möllendorff, 1888 by subsequent designation (Pilsbry 1895 in 1894–1895).

##### Diagnosis.

Shell small (H = 1.5 mm, W = 2.9 mm), helicoid, depressed or triangular, wider than high. Color uniformly brown or purplish. Apex prominent; protoconch smooth or with dense mesh of granular reticulation. Teleoconch smoothish, but with uneven, oblique striatae. Spire slightly concave, sometimes scalariform. Body whorl keeled or rounded, often with a groove below suture. Shell umbilicate; umbilicus narrow to moderately narrow. Aperture oblique, rounded, with 0–5 apertural teeth; peristome thin, expanded.

##### Remarks.

Páll-Gergely et al. (2019) suggested that Thai and Philippine *Aulacospira* may not be closely related due to their unusual distribution pattern and suggested that the similar shell shape of these snails may be due to convergence but more data are needed to support this hypothesis. However, we continue to use *Aulacospira* for Thai species following the description of this genus by Pilsbry (1916–1918).

#### Aulacospira depressa

Taxon classificationAnimaliaStylommatophoraHypselostomatidae

Dumrongrojwattana & Panha, 2006

C9C4D48E-143E-5A96-959D-B50917B6E82D

Figures 2A , 3A

Aulacospira depressus Dumrongrojwattana and Panha 2006: 121–122, fig. 2.

##### Remarks.

The specific epithet “*depressus*” must be corrected to “*depressa*” to agree in gender with *Aulacospira* (musculine).

##### Type locality.

Thailand, Khao Chakan, an isolated limestone hill of Srakeo Province; 13°48'02"N, 102°12'49"E; ca 85 m a.s.l.

##### Types examined.

**Figure 2.**
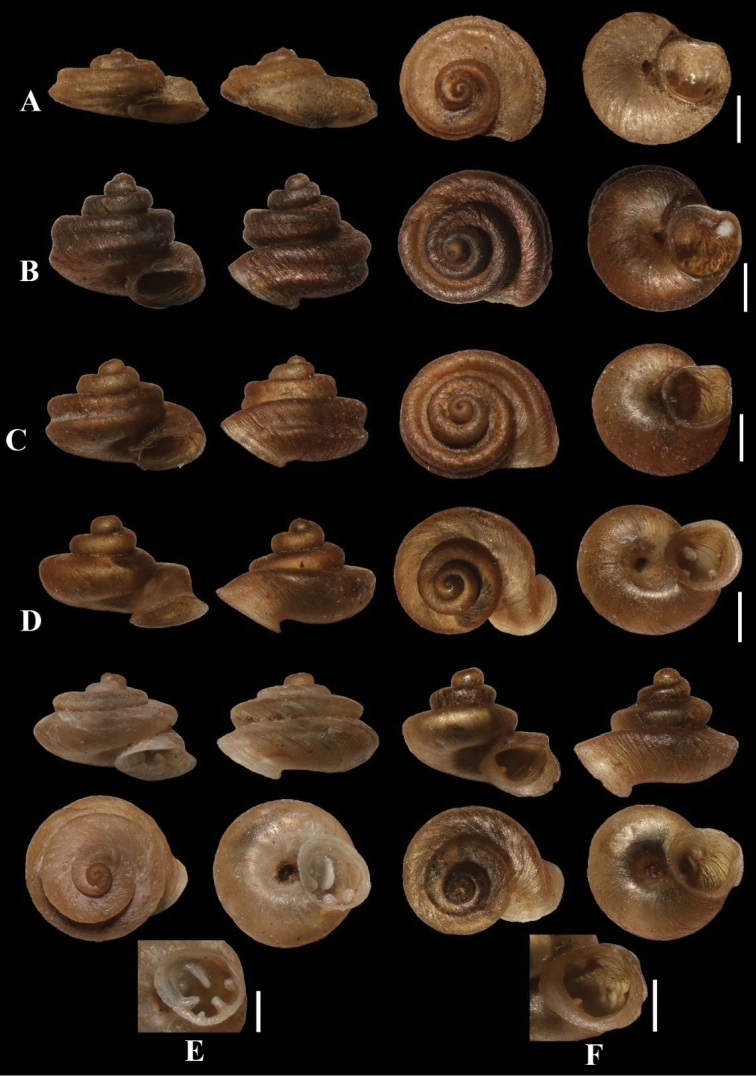
*Aulacospira* spp. **A***A. depressa* (paratype, BuUZM-MS 0077) **B***A. khaobote* (BuUZM-MS 0083) **C***A. khaopratun* (paratype, BuUZM-MS 0070) **D***A. lampangensis* (ZRCBUU 0403) **E***A. panhai* (paratype, ZRCBuU 0293) **F***A. smaesarnensis* (ZRCBUU 0370). Scale bars: 1 mm.

***Holotype*.**ZRCBUU 0076 (BuUZM-MS 0076) (Fig. 2A, B). ***Paratype*.**ZRCBUU 0077 (BuUZM-MS 0077).

##### Material examined.

ZRCBUU 0078 (BuUZM-MS 0078) (3 shells); Thailand, Khao Chakan, an isolated limestone hill of Srakeo Province; 13°48'02"N,102°12'49"E; ca 85 m a.s.l.; 8.vi.2003; leg. Dumrongrojwattana P. ZRCBUU 0096 (BuUZM-MS 0096) (24 shells); 19.iv.2003; leg. Dumrongrojwattana, P. ZRCBUU 0352 (10 shells); Thailand, Khao Chakan, an isolated limestone hill of Srakeo Province; 13°48'02"N,102°12'49"E, ca 85 m a.s.l.; 18.i.2020; leg. Dumrongrojwattana, P.

##### Measurements.

H = 0.97–1.14 mm, W = 2.51–3.15 mm.

##### Diagnosis.

Shell minute, very depressed; spire very low; brownish. Protoconch smooth; teleoconch smooth; body whorl large, with a prominent groove which forms two carinae; tuba very short; peristome expanded; aperture lacking teeth (Fig. 2A).

##### Radula.

**Figure 3.**
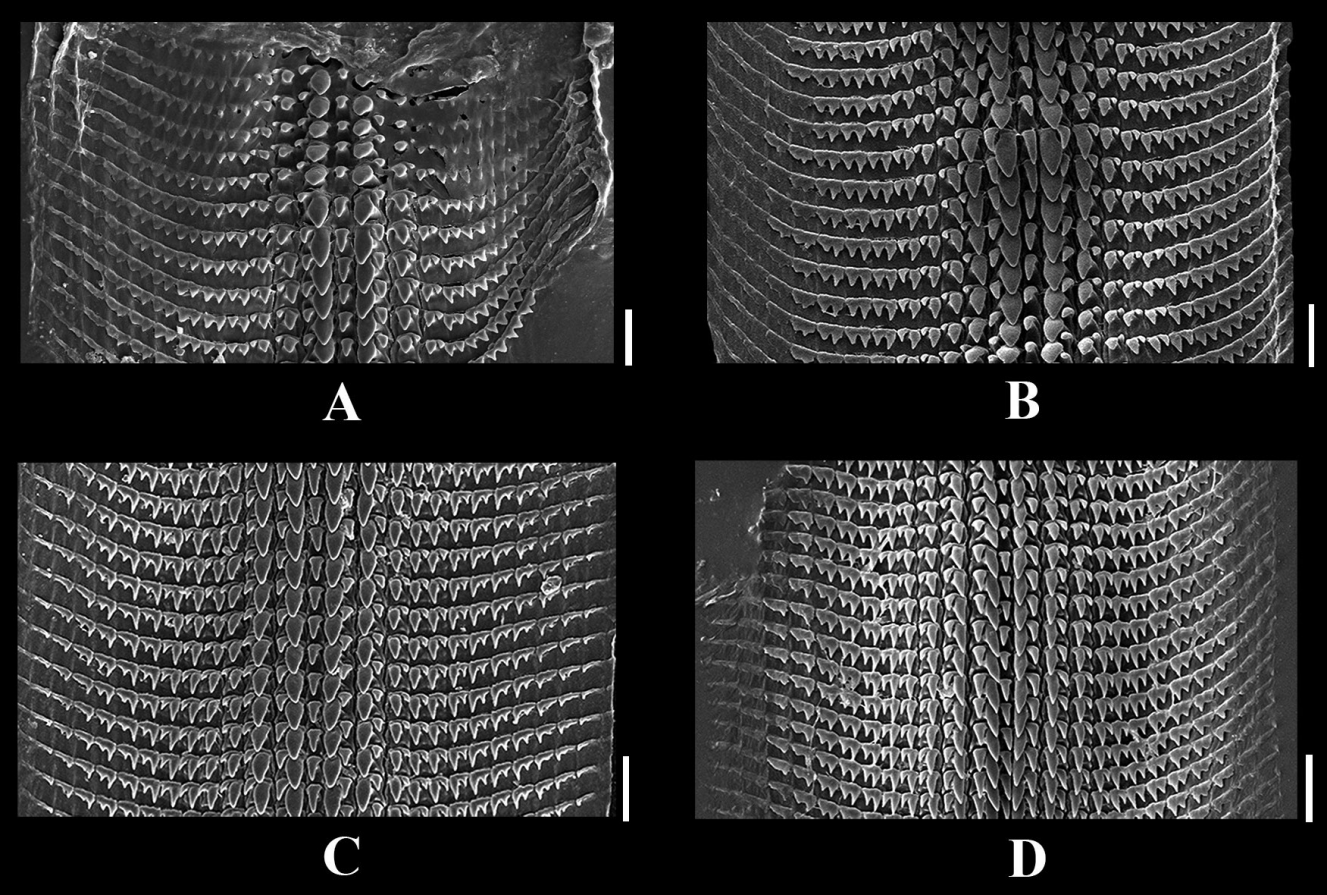
Radula of *Aulacospira* spp. **A***A. depressa***B***A. khaopratun***C***A. panhai***D***A. smaesarnensis.* Scale bars: 20 µm.

Central tooth small, unicuspid, triangular. Laterals irregularly bicuspid and consisting of a large internal cusp near and adjacent to a smaller, shorter outer cusp. Four laterals on each side of central tooth; first tooth largest, other teeth sequentially smaller. Marginals also irregularly, unequally bicuspid, with internal cusp larger than outer cusp. Marginal teeth 7 or 8 on each side of central tooth (Fig. 3A).

***Radula formula*.** 7–8:4:1:4:7–8.

##### Genital system.

Unknown.

##### Distribution.

This species is known only from the type locality (Fig. 1).

#### Aulacospira khaobote

Taxon classificationAnimaliaStylommatophoraHypselostomatidae

Dumrongrojwattana & Panha, 2006

386B7E93-AC88-5A24-9B6D-66C58A41FBBD

Figure 2B

Aulacospria khaobote Dumrongrojwattana and Panha 2006: 122–123, fig. 3.

##### Type locality.

Thailand, Wat Tam Khao Bote, an isolated limestone hill of Rayong Province; 13°09'19"N,101°38'05"E.

##### Types examined.

***Holotype*.**ZRCBUU 0083 (BuUZM-MS 0083) (Fig. 2G). ***Paratype*.**ZRCBUU 0084 (BuUZM-MS 0084).

##### Measurements.

H = 1.59–1.89 mm, W = 1.91–2.38 mm.

##### Diagnosis.

Shell minute, triangular; spire high; brownish. Protoconch smooth; teleoconch smooth; body whorl with broad sulcus; tuba very short; peristome not expanded; aperture lacking teeth.

##### Radula.

Unknown.

##### Genital system.

Unknown.

##### Distribution.

This species is known only from the type locality (Fig. 1).

#### Aulacospira khaopratun

Taxon classificationAnimaliaStylommatophoraHypselostomatidae

Dumrongrojwattana & Panha, 2005

619DEBD7-53FD-5342-B707-CB90CD5412E9

Figures 2C , 3B

Aulacospria khaopratun Dumrongrojwattana and Panha 2005: 15–16, fig. 2.

##### Type locality.

Thailand, Khaopratun, an isolated limestone hill of Rayong Province; 13°07'18"N,101°36'03"E.

##### Types examined.

***Holotype*.**ZRCBUU 0072 (BuUZM-MS 0072) (Fig. 2C, D). ***Paratype*.**ZRCBUU 0073 (BuUZM-MS 0073).

##### Material examined.

ZRCBUU 0298 (10 shells); Thailand, Khao Cha-ang Hayod, an isolated limestone hill of Chonburi Province; 13°09'40.6"N,101°35'51.4"E; 23.iii.2008; leg. Dumrongrojwattana, P. ZRCBUU 0350 (15 shells); Thailand, Subthaworn Temple, a place located in an isolated limestone hill of Srakeo Province; 13°24'19.5"N,102°16'29.8"E; 19.vi 2010; leg. Inmadon, R. ZRCBUU 0440 (12 shells); Thailand, Phet Pananikom Temple, a place located in an isolated limestone hill of Srakeo Province; 13°29'17.5"N,102°04'48.6"E; 12.vii. 2015; leg. Inmadon, R. ZRCBUU 0490 (12 shells); Phet Pananikom Temple, a place located in an isolated limestone hill of Srakeo Province; 13°29'17.5"N,102°04'48.6"E; 23.vi. 2016; leg. Inmadon, R.

##### Measurements.

H = 1.61–1.80 mm, W = 1.81–2.3 mm.

##### Diagnosis.

Shell minute, depressed; spire moderately high; brownish. Protoconch smooth; teleoconch smooth; body whorl large, with two prominent spiral carinae; tuba short; peristome expanded; aperture lacking teeth.

##### Radula.

As in *A. depressa* (Fig. 3B).

##### Genital system.

Unknown

##### Distribution.

Chonburi, Rayong, and Srakeo provinces, eastern Thailand (Fig. 1).

#### Aulacospira lampangensis

Taxon classificationAnimaliaStylommatophoraHypselostomatidae

Panha & Burch, 2002

7F0C8C27-6AD0-5163-A90D-FD0B70B366DB

Figure 2D

Aulacospira lampangensis Panha and Burch 2002: 70, fig. 3.

##### Type locality.

Thailand, Ban Thasee, an isolated limestone hill of Lampang Province; 18°25'18.8"N,99°45'11.6"E.

##### Material examined.

ZRCBUU 0403 (2 shells); Thailand, Ban Thasee, an isolated limestone hill of Lampang Province; 18°25'18.8"N,99°45'11.6"E; 3.vi. 2012; leg. Meesukko, C.

##### Measurements.

H = 1.6–1.8 mm, W = 2.0–2.3 mm.

##### Diagnosis.

Shell minute, depressed, with rounded whorls; spire moderately high; brownish. Protoconch smooth; teleoconch smooth; body whorl large, with two prominent spiral carinae; tuba projecting downward; peristome expanded; aperture with five teeth, columellar, parietal lamellae, upper and lower palatal plicae, and basal plica (Fig. 2D).

##### Radula.

Unknown.

##### Reproductive anatomy.

Unknown.

##### Distribution.

This species appears limited to the type locality (Fig. 1).

#### Aulacospira panhai

Taxon classificationAnimaliaStylommatophoraHypselostomatidae

Dumrongrojwattana, 2008

379DF856-5A94-5DFC-B223-38E20F450FF9

Figures 2E , 3C

Aulacospira panhai Dumrongrojwattana 2008: 57–59, fig. 1.

##### Type locality.

Thailand, Khaopratun, an isolated limestone hill of Rayong Province; 13°07'19"N, 101°36'03"E.

##### Types examined.

***Holotype*.**ZRCBUU 0220. ***Paratype*.**ZRCBUU 0293.

##### Material examined.

ZRCBUU 0353 (3 shells); Thailand, Khao Cha-ang Hayod, an isolated limestone hill of Chonburi Province; 13°09'40.6"N, 101°35'51.4"E; 31.i.2013; leg. Dumrongrojwattana, P. ZRCBUU 0393 (5 shells); Thailand, Khao Cha-ang Hayod, an isolated limestone hill of Chonburi Province; 13°09'40.6"N, 101°35'51.4"E; 23.vi.2014; leg. Dumrongrojwattana, P. ZRCBUU 0394 (2 shells); Thailand, Khaopratun, an isolated limestone hill of Rayong Province; 13°07'19"N, 101°36'03"E; 31.i.2013; leg. Dumrongrojwattana, P. ZRCBUU 0495 (2 shells); Thailand, Khao Cha-ang Hayod, an isolated limestone hill of Chonburi Province; 13°09'40.6"N,101°35'51.4"E; 15. x. 2016, leg. Dumrongrojwattana, P.

##### Measurements.

H = 2.47–2.83 mm, W = 1.45–1.70 mm.

##### Diagnosis.

Shell minute, semi-depressed; spire distorted; brownish. Protoconch granulose; teleoconch smooth; the first two whorls slightly flatten; the last two whorls large and inflated; tuba short and downwardly directed; peristome expanded; aperture with six teeth, parietal and infraparietal lamellae, upper palatal and lower palatal and basal plicae, and columella lamellae (Fig. 2E).

##### Radula.

As in *A. depressa* (Fig. 3C).

##### Reproductive anatomy.

Unknown.

##### Distribution.

Chonburi and Rayong provinces, eastern Thailand (Fig. 1).

#### Aulacospira smaesarnensis

Taxon classificationAnimaliaStylommatophoraHypselostomatidae

Panha & Burch, 2001

382705FE-8AE1-56A7-999C-1D632CDF793E

Figures 2F , 3D

Aulacospira smaesarnensis Panha and Burch 2001: 65, fig. 2.

##### Type locality.

Thailand, Smaesarn Village, a fishery village located in an isolated liestone hill of Chonburi Province; 12°34'06"N, 100°56'578"E.

##### Types examined.

ZRCBUU 0370 (10 shells); Thailand, Smaesarn Village, a fishery village located in an isolated liestone hill of Chonburi Province; 12°34'06"N, 100°56'578"E; 12.v.2013; leg. Dumrongrojwattana, P. ZRCBUU 0425 (10 shells); Thailand, Smaesarn Village, a fishery village located in an isolated limestone hill of Chonburi Province; 12°34'06"N, 100°56'578"E; 20.iii.2015; leg. Dumrongrojwattana, P.

##### Measurements.

H = 2.47–2.83 mm, W = 1.45–1.70 mm.

##### Diagnosis.

Shell minute, helicoid, moderately elevated spire; brownish. Protoconch smooth; teleoconch rough; body whorl large, rounded peripherally; tuba projecting downward; peristome thickened and expanded; aperture with poorly developed barriers, parietal lamella palatal plica and columellar lamella (Fig. 2F).

##### Radula.

As in *A. depressa* (Fig. 3D).

##### Reproductive anatomy.

Unknown.

##### Distribution.

This species appears limited to the type locality (Fig. 1).

#### Aulacospira pluangtong

Taxon classificationAnimaliaStylommatophoraHypselostomatidae

Panha & Burch, 2004

9361F0F9-720B-5B22-B18F-25706ED47247

Figure 4A–D

Aulacospira pluangtong Panha and Burch 2004: 64, fig. 5.

##### Type locality.

Thailand, Pluangtong Mountain, an isolated limestone hill of Chonburi Province; 13°11'50"N, 101°34'49"E.

##### Material examined.

ZRCBUU 357 (3 shells); Thailand, Khao Mee Mountain, an isolated limestone hill of Chonburi Province; 13°09'02.7"N, 101°35'34.4"E; 15.x.2016; leg. Dumrongrojwattana, P.

##### Measurements.

H = 1.75–1.82 mm, W = 1.82–1.93 mm.

##### Diagnosis.

Shell minute; spire high; brownish. Protoconch smooth; teleoconch smoothish, with uneven growth striae. Final part of the last whorl free and projecting downward; peristome not expanded; aperture with four teeth, parietal and colummellar lamellae, and the upper and lower palatal plica.

##### Radula.

**Figure 4.**
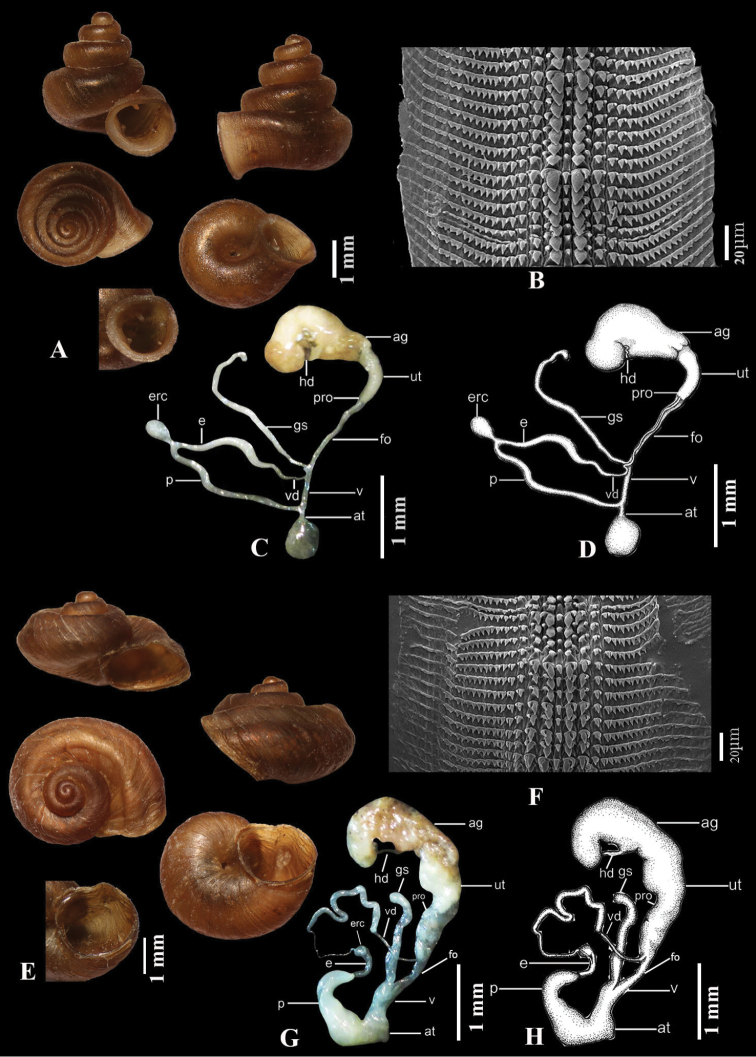
**A–D***Aulacospira pluangtong* (ZRCBUU 357) **A** shell **B** radula **C, D** genital system **E–H***Aulacospira nutadhirai* sp. nov. **E** holotype (ZRCBUU 0391) **F** radula **G, H** genital system.

As in *A. depressa* (Fig. 4B).

##### Genital system.

Atrium shorter than vagina. Penis longer than epiphallus, with anterior portion a slender tube. Epiphallus connected to distal end of penis. Epiphallus glossy white, longer than vas deferens, with its anterior portion slender and cylindrical, its central portion slender and more bulging than posterior portion. Epiphallic flagellum absent. Epiphallic retractor caecum rounded and bulbous, attached to posterior portion of epiphallus. Vas deferens very long, slender, entering epiphallus apically. Vagina and free oviduct cylindrical, with vagina shorter than free oviduct. Gametolytic sac very long and cylindrical, with anterior portion connecting vagina and free oviduct and posterior portion with curved knob. Uterus long and cylindrical, with very thin prostate gland adhering to it. Hermaphroditic duct loosely convolute. Albumen gland large and yellowish. Dart apparatus absent (Fig. 4C, D).

##### Distribution.

This species is only known from the limestone hills in Botong District, Chonburi Province, eastern Thailand (Fig. 1).

#### Aulacospira nutadhirai sp. nov.

Taxon classificationAnimaliaStylommatophoraHypselostomatidae

BE09AD04-3023-5F80-B7C0-D2C5641A7B6A

http://zoobank.org/5FE507E0-15A0-4537-B3E3-429D95103C90

Figure 4E–H

##### Types examined.

***Holotype*.**ZRCBUU 0391; Thailand, Khao Thanan, an isolated limestone hill of Thung-wa District, Satun Province; 7°03'42.2"N, 99°41'32.9"E; 10.iv.2010; leg. Dumrongrojwattana, P. ***Paratypes*.**ZRCBUU 0392 (10 shells); 10.iv.2010; ZRCBUU 0420 (5 shells); THNHM-iv-18802 (2 shells); MNHN IM-2014-7121 (2 shells); NHLRU 0010 (2 shells); collected from location same as holotype, 27.iv.2015; leg. Dumrongrojwattana, P.

##### Measurments.

Holotype H = 1.54 mm, W = 2.89 mm. Paratypes H = 1.47–2.50 mm, W = 2.61–3.07 mm.

##### Diagnosis.

Shell minute, helicoid. Protoconch smooth; body whorl stout, with a short projecting downward tuba; peristome not expanded; aperture lacking teeth.

*Aulacospira nutadhirai* sp. nov. is very similar to the eastern Thai species, *A. khaopratun*, but differs in its lower spire and more greatly inflated last whorl. Compared to Philippines species, *Aulacospira nutadhirai* sp. nov. is similar to *A. porrecta* but differs in the shape of the shell and in having no keel on the body whorl.

##### Description.

Shell minute, helicoid, brownish, with 4–4½ whorls. Body whorl stout. Last quarter of body whorl with a short tuba projecting downward. Protoconch, consisting of 1¼ whorls, with granulose wrinkles. Teleoconch smoothish, with uneven oblique growth striae. Suture deep. Shell narrowly umbilicate. Spire low; first two whorls rounded. Penultimate whorl and body whorl with two shallow spiral sulci that continuously to peristome. Peristome expanded; aperture round and lacking teeth (Fig. 4E).

##### Radula.

As in *A. depressa* (Fig. 4F).

##### Genital system.

Atrium shorter than vagina. Penis shorter than epiphallus, with anterior and central portion large, bulging and posterior portion curved. Epiphallus connected to distal end of penis. Epiphallus glossy white, longer than vas deferens, with anterior portion cylindrical, central and posterior portion cylindrical, and distal end curved. Epiphallic flagellum absent. Epiphallic retractor caecum rounded, connected to distal part of epiphallus. Vas deferens short, slender, entering epiphallus apically. Vagina and free oviduct cylindrical, with vagina large and shorter than free oviduct. Gametolytic sac long and cylindrical, with anterior portion connecting vagina and free oviduct, posterior portion swollen. Uterus long, large, with very thin prostate gland adhering to it. Hermaphroditic duct loosely convolute. Albumen gland large and yellowish. Dart apparatus absent (Fig. 4G, H).

##### Type locality.

Thailand, Khao Thanan, an isolated limestone hill of Thungwa District, Satun Province; 7°03'42.2"N, 99°41'32.9"E.

##### Etymology.

We name this species in honor of Mr Thammarat Nutathira or Kru Nok, a former staff member of Kampang Wittaya School, Thailand, who contributed to the study of the biodiversity and paleontology of the limestone in Satun Province.

##### Distribution.

This species appears limited to the type locality (Fig. 1).

#### Aulacospira tekavongae sp. nov.

Taxon classificationAnimaliaStylommatophoraHypselostomatidae

E8975E6F-0CC8-5CC0-B8B8-2ACDE718AB61

http://zoobank.org/7572347F-FC91-4E19-BC65-748541761EE1

Figure 5A–D

##### Type material.

***Holotype*.**ZRCBUU 0610; Thailand, Khao Chakan, an isolated limestone hill of Srakeo Province; 240 m a.s.l.; 13°48'02"N, 102°12'49"E; 5.v.2019; leg. Kamtuptim, C. and Dumrongrojwattana, P. ***Paratypes*.**ZRCBUU 0611 (15 shells); ZRCBUU 0420 (10 shells); 19.v.2014; leg. Dumrongrojwattana, P. THNHM-Iv-18803 (5 shells); MNHN IM-2014-7122 (5 shells); 5.v.2019; location same as holotype; leg. Kamtuptim, C. and Dumrongrojwattana, P.

##### Measurments.

Holotype H = 2.32 mm, W = 2.08 mm. Paratypes H = 1.94–2.35 mm, W = 1.95–2.21 mm.

##### Diagnosis.

Shell minute, conical. Protoconch smooth, body whorl with a very short projecting downward tuba; peristome expanded; aperture lacking teeth.

*Aulacospira tekavongae* sp. nov. is very similar to *A. khaobote*, but the shell is a conical and with a high spire, while that of *A. khaobote* shell helicoid and with a low spire.

##### Description.

Shell minute, conical, brownish, with 4–4½ whorls. Tuba very short, projecting downward. Protoconch consisting of 1 ¼ whorls, with granulose wrinkles. Teleoconch smoothish, sculptured with uneven, oblique growth striae. Suture deep. Shell narrowly umbilicate. Spire high; first two whorls rounded, penultimate and body whorl with two distinct spiral sulci continuously to peristome. Peristome expanded; aperture round and lacking teeth (Fig. 4A).

##### Radula.

**Figure 5.**
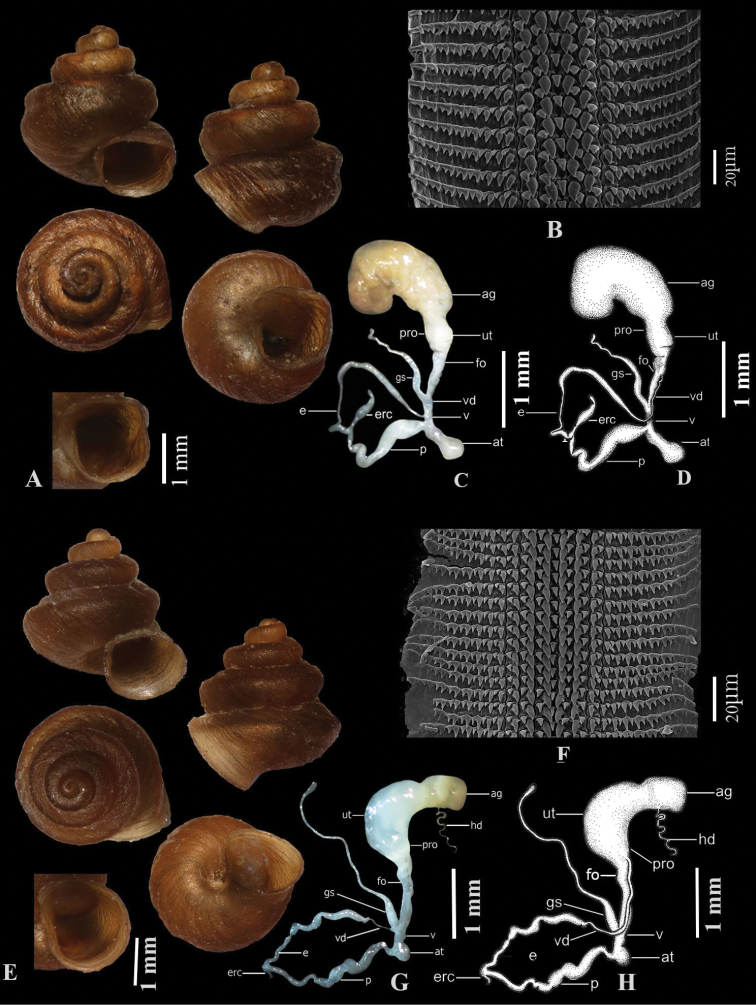
**A–D***Aulacospira tekavongae* sp. nov. **A** holotype (ZRCBU 0610) **B** radula **C, D** genital system **E–H***Aulacospira vanwalleghemi* sp. nov. **E** holotype (ZRCBUU 732) **F** radula **G, H** genital system.

As in *A. depressa* (Fig. 5B).

##### Genital system.

Atrium longer than vagina. Penis shorter than epiphallus, with anterior portion a short tube and bulging. Epiphallus connected to distal end of penis. Epiphallus longer than vas deferens, with anterior portion slender and cylindrical, central portion slender and more bulging than anterior and posterior portion. Epiphallic flagellum absent. Epiphallic retractor caecum rather bulging, attached to posterior portion of epiphallus. Vas deferens long, slender, entering epiphallus apically. Vagina and free oviduct cylindrical, with vagina shorter than free oviduct. Gametolytic sac long and cylindrical, with anterior and central portion bulging, posterior portion slender and curved knob. Uterus long and large, with very thin prostate gland adhering to it. Hermaphroditic duct loosely convolute. Albumen gland large and yellowish. Dart apparatus absent (Fig. 5C, D).

##### Type locality.

Thailand, Khao Chakan, an isolated limestone hill of Srakeo Province, eastern Thailand; 13°48'02"N, 102°12'49"E; ca 240 m a.s.l.

##### Etymology.

We name this species in the hornor of Ms Rattanawadee Tekavong, a research collaborator, who has worked extensively on the eastern microsnail diversity.

##### Distribution.

This species is known only from the type locality (Fig. 1).

#### Aulacospira vanwalleghemi sp. nov.

Taxon classificationAnimaliaStylommatophoraHypselostomatidae

CB33BD03-55B3-5853-9033-4FA8D73F8773

http://zoobank.org/1683A4B8-98CB-40C0-A5BB-E720DAD400C8

Figure 5E–H

##### Types examined.

***Holotype*.**ZRCBUU 0732; Thailand, Wat Khao Tam Ratt, an isolated limestone hill of Ta Takeab District, Chacheongsao Province; ca 35 m a.s.l.; 13°23'30.4"N, 101°44'39.1"E; 19.i.2020; leg. Dumrongrojwattana, P. ***Paratype*.**ZRCBUU 0733 (8 shells); as in holotype.

##### Measurments

**(in mm).** Holotype H = 3.17 mm, W = 3.28 mm. Paratypes H = 1.87–2.63 mm, W = 1.74–2.57 mm.

##### Diagnosis.

Shell minute, conical. Protoconch smooth; body whorl peripherally with a very with a very short projecting downward tuba; peristome expanded; aperture round and lacking teeth.

The shell shape and periphery of the last whorl of *A. vanwalleghemi* sp. nov. is similar to *A. pluangtong*, but apertural teeth are absent. Apertural teeth are present in *A. pluangtong*.

##### Description.

Shell minute, conical, brownish, with 4–4½ whorls. Tuba very short, projecting downward. Protoconch consists of 1¼ whorls, granulosely wrinkled. Teleoconch smoothish, sculptured with uneven, oblique growth striae. Suture deep. Shell narrowly umbilicate. Spire high, with peripherally of most whorls rounded; penultimate and body whorls with strong keel at periphery continuously to peristome. Peristome expanded; aperture obliquely oval and lacking teeth (Fig. 5E).

##### Radula.

As in *A. depressa* (Fig. 5F)

##### Genital system.

Atrium shorter than vagina. Penis shorter than epiphallus, with anterior and central portion large, bulging and posterior portion curved. Epiphallus connected to distal end of penis. Epiphallus longer than vas deferens, with anterior portion with cylindrical, central and posterior portion cylindrical, distal end curved; white glossy. Epiphallic flagellum absent. Epiphallic retractor caecum rounded, connected to distal part of epiphallus. Vas deferens short, slender, entering epiphallus apically. Vagina and free oviduct cylindrical, with vagina large and shorter than free oviduct. Gametolytic sac a very long and slender, with anterior portion bulging, connects between vagina and free oviduct, posterior portion curved. Uterus long and large, with very thin prostate gland adhering to it. Hermaphroditic duct loosely convolute. Albumen gland large and yellowish. Dart apparatus absent (Fig. 5G, H).

##### Type locality.

Thailand, Wat Khao Tam Ratt, an isolated limestone hill of Ta Takeab District, Chacheongsao Province; 13°23'30.4"N, 101°44'39.1"E; ca 35 m a.s.l.

##### Etymology.

We name this species in honor of Mr René Vanwalleghem, a Belgian conchologist who inspired the senior author to pursue mollusc research.

##### Distribution.

This species appears limited to the type locality (Fig. 1).

### Key to species of *Aulacospira* in Thailand

This key is based on shell morphogy and is modified from Panha and Burch (2005).

**Table:** 

1	Shell without spiral carinae; apertural teeth present	**2**
–	Shell with or without spiral carinae; apertural teeth absent	**5**
2	Shell depressed or conical-shaped; aperture with 3 or 4 barriers	**3**
–	Shell globular; spire distorted; body whorl with peripheral angle; 6 apertural teeth present	***Aulacospira panhai*** (Fig. 6E)
3	Spire depressed or high; apertureed with 4 barriers	**4**
–	Spire moderately high; aperture with 3 barriers: parietal lamella, lower palatal plica and columella lamella	***A. smaesarnensis*** (Fig. 6G)
4	Spire depressed; body whorl shouldered; aperture with 4 barriers: parietal lamella, upper and lower palatal plica and columella lamella	***A. lampangensis*** (Fig. 6D)
–	Spire high; body whorl obtusely angular; aperture with 4 barriers, parietal lamella, upper and lower palatal plica and columella lamella	***A. pluangtong*** (Fig. 6F)
5	Shell conical-shaped, spire high body whorl with or without spiral groove	**6**
–	Shell depressed, body whorl with spiral groove	**7**
6	Body whorl peripherally keeled	***A. vanwalleghemi*** (Fig. 6J)
–	Body whorl with spiral groove	***A. tekavongae*** (Fig. 6I)
7	Spire high or moderately high; body whorl with deep spiral groove	**8**
–	Spire low, body whorl with deep or shallow spiral groove	**9**
8	Spire moderately high, W/H = ca 1.71	***A. khaopratun*** (Fig. 6C)
–	Spire high, W/H = ca 1.21	***A. khaobote*** (Fig. 6B)
9	Shell very flattened; body whorl with deep spiral groove; shell	***A. depressa*** (Fig. 6A)
–	Shell stout; body whorl with shallow spiral groove	***A. nutadhira*** (Fig. 6H)

**Figure 6.**
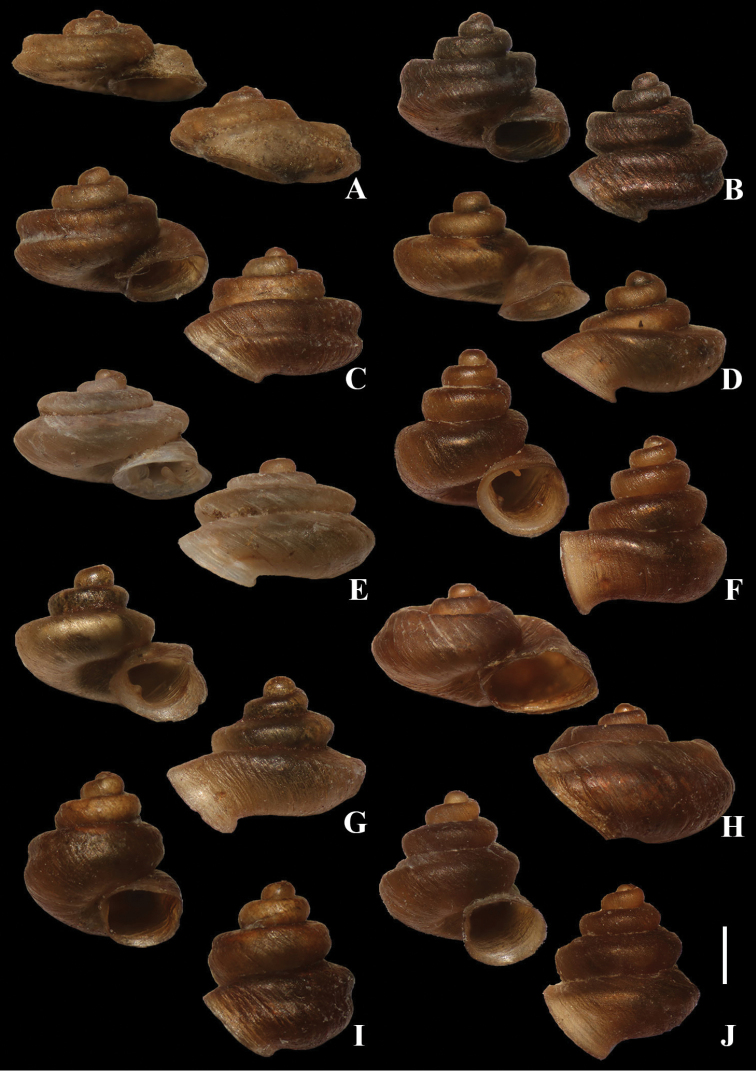
Synoptic view of *Aulacospira* Möllendorff, 1890 in Thailand **A***A. depressa***B***A. khaobote***C***A. khaopratun***D***A. lampangensis***E***A. panhai*. **F***A. pluangtong***G***A. smaesarnensis***H***Aulacospira nutadhirai* sp. nov. **I***Aulacospira tekavongae* sp. nov. **J***Aulacospira vanwalleghemi* sp. nov. Scale bars: 1 mm.

## Discussion

All species recorded here can be categorized into two groups based on shell morphology: 1) apertural teeth present and no conspicuous groove on the body whorl, and 2) the apertural teeth absent and with a conspicuous groove on the body whorl. The group with apertural teeth and without a conspicuous groove on the body whorl is composed of *A. lampangensis*, *A. panhai*, *A. pluangtong*, and *A. smaesarnensis*, while the other group without apertural teeth and with a conspicuous groove on the body whorl comprises *A. depressa*, *A. khaobote*, *A. khaopratun*, *A. nutadhirai* sp. nov., and *A. tekavongae* sp. nov. *Aulacospira vanwalleghemi* sp. nov. cannot be classified into either of these groups because both the apertural barrier and conspicuous groove are absent. The shell shape of *A. panhai* is distinctively different from other Thai species; its streptaxoid shell shape resembles the genus *Pseudostreptaxis* Mölendorff, 1890 and especially *P. azpeitiae* (Hidago, 1890) from the Philippines, which was described only by shell morphology (Möllendorff 1890; Páll-Gergely et al. 2019). We place *A. panhai* in *Aulacospira*, but more data, including anatomical and molecular, are needed to clarify its taxonomic placement.

The radulae of all examined species are tongue-shaped and with about 23–25 teeth per row. The radula formula is 7–8:4:1:4:7–8; there is a unicuspid central, larger bicuspid laterals, and a small, unequal bicuspid marginal. Compared with the microsnail genus *Pupoides* L. Pfeiffer, 1854 (Pupillidae Turton, 1831) there are 32 radula teeth per row, with the centrals trifid, laterals bifid, and marginal multicuspid. (Branson 1961) but in this study, the number of teeth per row in *Aulacospira* is fewer and shape of the teeth differs.

In this study, four species of the genus *Aulacospira* are described, based on characters of shells and genital systems. *Aulacospira pluangtong* was found to differ from other newly named species of the genus by its penis, vas deferens, and gametolytic sac. The penis of *A. pluangtong* is longer than the epiphallus and the vas deferens is a very long compared with the other new species.

Eastern Thailand and especially Chonburi and Rayong provinces seem to be a diversity hot spot for this genus and all species show high endemism (Fig. 1; Table 1). Páll-Gergely et al. (2019) remarked on the unusual geographic distribution of *Aulacospira* split between the Philippines and Thailand. Further studies, including over a broader geographical area and molecular analyses, are needed to explain the biogeography of this genus.

## Supplementary Material

XML Treatment for Aulacospira

XML Treatment for Aulacospira depressa

XML Treatment for Aulacospira khaobote

XML Treatment for Aulacospira khaopratun

XML Treatment for Aulacospira lampangensis

XML Treatment for Aulacospira panhai

XML Treatment for Aulacospira smaesarnensis

XML Treatment for Aulacospira pluangtong

XML Treatment for Aulacospira nutadhirai

XML Treatment for Aulacospira tekavongae

XML Treatment for Aulacospira vanwalleghemi
